# Association of genetic variations in FOXO3 gene with susceptibility to noise induced hearing loss in a Chinese population

**DOI:** 10.1371/journal.pone.0189186

**Published:** 2017-12-08

**Authors:** Haoran Guo, Enmin Ding, Ying Bai, Hengdong Zhang, Huanxi Shen, Jun Wang, Xianping Song, Wenyan Cai, Jiadi Guo, Baoli Zhu

**Affiliations:** 1 School of Public Health, Southeast University, Nanjing, Jiangsu Province, China; 2 Institute of Occupational Disease Prevention, Jiangsu Provincial Center for Disease Prevention and Control, Nanjing, Jiangsu Province, China; 3 Kunshan Centers for Disease Prevention and Control, Kunshan, Jiangsu Province, China; 4 School of Public Health, Nanjing Medical University, Nanjing, Jiangsu Province, China; Duke Cancer Institute, UNITED STATES

## Abstract

Noise induced hearing loss (NIHL), a multifactorial disease involving both genetic and environmental factors, is one of the most important occupational health hazards. Nonetheless, the influence of FOXO3 variants on NIHL risk have not been illuminated. This research was conducted to explore the effects of FOXO3 polymorphisms on individual susceptibility to NIHL. A total of 2689 industrial workers from one textile factory of east China were recruited to participate in the current research. Venous blood was collected, questionnaire and pure-tone audiometry (PTA) was conducted by specialist physicians. Then, we performed genotyping of three selected SNPs (rs2802292, rs10457180, and rs12206094) in FOXO3 gene in 566 NIHL patients and 566 controls. Subsequently, the main effects of genotype and its interactions were evaluated. Our results revealed that individuals with the G allele of rs2802292, G allele of rs10457180, T allele of rs12206094 (OR = 1.43, 1.43, and 1.31 respectively) and the haplotype GAC and others (TGT/GGT/GGC/GAT) (rs2802292-rs10457180-rs12206094) (OR = 1.49 and 2.09 respectively) are associated with an increased risk of NIHL in a Chinese population. Stratified analysis showed that an increased NIHL risk was found in the subjects who exposed to noise >16 years with rs2802292 GG/GT and rs10457180 AG/GG genotype with an OR of 1.62 and 1.66 respectively. Multifactor dimensionality reduction analysis indicated that rs10457180, rs2802292, and rs12206094 have interactions and are related to increased NIHL risk (OR = 1.53). The genetic polymorphism rs2802292, rs10457180, and rs12206094 within FOXO3 gene are associated with an increased risk of NIHL in a Chinese population and have potential to be biomarkers for noise exposed workers.

## Introduction

Occupational noise is one of the most common occupational hazards for the health of industrial workers, and noise induced hearing loss (NIHL) is the second most frequent form of sensorineural hearing impairment besides age-related hearing loss (ARHL) worldwide[1]. It has been verified that NIHL is a kind of multifactorial disease resulting from the interactions of both genetic and environmental factors[2]. Currently, the mechanism of NIHL has not been completely understood. The possible etiopathogenesis may involve the inner ear cell apoptosis or necrosis caused by oxidative stress or the metabolic products generated during signal transduction and direct mechanical injury to the structures of the cochlea[3–5]. However, numerous population studies have indicated that the subjects had various degrees of NIHL risk even if they were exposed to equal noise intensity level[1, 6]. Animal experiments also prove that genetic variations contribute to the incidence of NIHL[7, 8]. Previous studies have found that single nucleotide polymorphism (SNPs) in HSP70, EYA4, CDH23, GRHL2 and DFNA5 genes are associated with human genetic susceptibility to NIHL and could increase or decrease the risk of NIHL by interaction with occupational noise[9–11]. All the evidence implicates that the genetic susceptibility and its interaction with environmental factors might play an important role in the occurrence and development of NIHL.

Forkhead Box O3 (FOXO3) is a winged helix transcription factor that has been known to regulate longevity in various species including humans and mice[12–14]. FOXO3 regulates the expression of stress response proteins[15] and FOXO3 effectors may ameliorate oxidative stress, block mitosis, induce apoptosis, or promote inflammation[16]. It has been shown that mice are susceptible to adult-onset hearing loss with the hallmark characteristics of auditory neuropathy, namely, elevated auditory thresholds combined with normal outer hair cell function if lacking the transcription factor Foxo3[17]. Also, Foxo3 was found to be necessary for auditory function after noise exposure in a mouse model system and outer hair cells are lost throughout the middle and higher frequencies in absence of Foxo3[18].

However, associations between NIHL and FOXO3 SNPs and their functional significance in the FOXO3 gene were not reported before. In consideration of the vital functions of FOXO3 in hearing maintenance, we speculated that the polymorphisms in FOXO3 gene might have associations with the genetic susceptibility to NIHL. Herein, a case-control study was performed to elucidate the associations between three FOXO3 SNPs, namely rs2802292, rs10457180, and rs12206094, with the genetic susceptibility of NIHL.

## Materials and methods

### Subjects

The subjects of the current study were all industrial employees from one textile factory of east China who received annual health examinations which were performed by the Jiangsu Provincial Center for Disease Prevention and Control in 2015. A total amount of 2689 people participated in health examinations. Before the investigation, informed consent was obtained from each participant. This study was approved by the Institutional Review Board of Jiangsu Provincial Center for Disease Prevention and Control. Occupational health examination items mainly included venous blood collection, general physical examination and pure-tone audiometry (PTA). During the health examination, personal medical history, smoking, drinking habits and habitual use of drugs were queried. Subjects were excluded from this study according to the following criterions: *a)* workers with diseases that may affect hearing thresholds (e.g. otitis media, cholesteatoma, ear canal stenosis, etc.); *b)* workers who have used or are using ototoxic drugs (e.g. aspirin, quinolones, aminoglycosides, etc.). Eventually, 2477 subjects conformed to our criterions.

### PTA and NIHL assessment

Audiometry was performed for each participant only after stopping noise exposure for up to12 hours. By using a Madsen Voyager 522 audiometer (Madsen, Taastrup, Denmark), pure tone audiometry was conducted by qualified doctors in a soundproof room.

### Definitions of NIHL and control subjects

Hearing loss and normal hearing are identified according to China diagnostic criteria of occupational noise induced deafness (GBZ 49–2007). In this study, occupational noise exposure is identified as levels of noise exposure (Lex) are at least 85 dB (A) for a nominal 8-hour working day. Hearing loss was identified as the binaural hearing thresholds which exceed 25 dB at both high (3000, 4000, 6000 Hz) and speech (500, 1000, 2000 Hz) frequencies. Correspondingly, normal hearing means that the binaural hearing thresholds are under 25 dB both at high and speech frequencies. Hearing thresholds were obtained from the results of PTD. The subjects were divided into two groups: NIHL patient group (noise exposed individuals with hearing loss) and control group (noise exposed individuals with normal hearing). We first selected the NIHL patients, and controls were frequency-matched to the patients by sex, age, and intensity of noise exposure [19]. Eventually, 566 NIHL patients and 566 controls were selected from all eligible subjects.

### DNA extractions

Peripheral blood (3 mL) was collected in ethylene diamine tetra acetic acid (EDTA) and taken for DNA isolation and genotyping. DNA was extracted from blood samples of subjects by using the QIAcube HT and QIAamp 96 DNA QIAcube HT Kit (Qiagen, Dusseldorf, Germany) following the manufacturer’s protocol and then stored at -20°C until use.

### SNP selection and genotyping

Target SNPs in the FOXO3 genes were selected on the basis of the 1000 Genomes Project database and previous findings from the literature. The criteria for searching for SNPs were as follows: *a)* MAF (minor allele frequency) of CHB > 0.10; *b)* a linkage disequilibrium value of r^2^ >0.8. SNPs of which DNA sequence are not suitable for PCR primer design were excluded. In the end, eleven candidate SNPs were selected using these criteria by Haploview software. Then, we searched these candidate SNPs in Pubmed and found that rs2802292, rs10457180 and rs12206094 were the most commonly reported SNPs of FOXO3 in previous findings. Ultimately three SNPs in the FOXO3 gene were selected for genotyping: rs2802292, rs10457180, and rs12206094.

Genotypes for the selected polymorphisms were screened with the ABI TaqMan SNP genotyping assays (Applied Biosystems, Foster City, Calif., USA) by using the pre-designed commercial genotyping assays. The extracted DNA and genotyping assays were added to TaqMan universal PCR master mix (Roche, Branchburg, N.J., USA) according to the manufacturer’s instructions. The genotyping procedures were then performed by ABI 7900 real-time PCR system (Applied Biosystems). The results were analyzed using ABI 7900 System sequence detection software version 1.2.3 (Applied Biosystems).

### Statistical analyses

Statistical analyses was performed by using SPSS 23.0 software (Chicago, IL, USA). The goodness-of-fit χ^2^ tests were conducted for the Hardy-Weinberg equilibrium rule of the SNPs in FOXO3 gene among the control subjects. Categorical variables are represented as percentages and continuous variables are described as the mean ± SD. Odds ratios (ORs) and 95% confidence interval (95%CI) for genotypes were achieved under conditional logistic regression models adjusted for age, sex, smoking, and drinking. Differences in allele-specific promoter activity and gene expression were compared by Student’s t-test or paired t-test. Haplotype analysis of the polymorphisms was performed using the SHEsis platform [20]. All the *P* values were corrected (*P*_*c*_) with Bonferroni corrections and *P* < 0.05 was used as the criterion of statistical significance.

## Results

### Demographic characteristics of study subjects and Hardy-Weinberg test of selected SNPs

The general characteristics (age, sex, smoking, drinking, work time with noise and exposure level with noise) and high-frequency hearing threshold of the NIHL cases and controls were shown in **Table 1**. No significant differences in general characteristics were shown between NIHL cases and controls (*P* > 0.05). Results also show that there was statistically significant difference between NIHL cases and controls in terms of high-frequency hearing threshold. The average high-frequency hearing threshold was higher for patients (35.77± 9.86) than for controls (14.02± 4.17) (*P* < 0.001). General information of selected SNPs and results of Hardy-Weinberg test were shown in **Table 2**. Rs2802292, rs10457180, and rs12206094 are located in 5’ UTR variant, intron variant, intron variant (upstream variant 2KB) of FOXO3 gene respectively and χ^2^ tests revealed that all SNPs were in the balance of Hardy-Weinberg (*P*>0.05).

**Table 1:** Demographic characteristics of study subjects.

Variables	Cases (n = 566)		Controls (n = 566)		*P*
	n	%	n	%	
Age (years)					0.879^a^
Mean±SD	40.52±6.26		40.31±5.85		0.556^b^
≤ 35	133	23.5	138	24.4	
35–45	320	56.5	321	56.7	
> 45	113	20.0	107	18.9	
Sex					0.646^a^
Male	527	93.1	523	92.4	
Female	39	6.9	43	7.6	
Smoking					0.263^a^
Now	331	58.5	315	55.7	
Ever	11	1.9	19	3.4	
Never	224	39.6	232	41.0	
Drinking					0.898^a^
Now	233	41.2	235	41.5	
Ever	10	1.8	12	2.1	
Never	323	57.1	319	56.4	
Work time with noise (years)					0.237^a^
Mean±SD	18.69±7.59		18.06±7.03		0.145^b^
≤ 16	248	43.8	261	46.1	
> 16	318	56.2	305	53.9	
Expose level with noise (dB)					0.712^a^
Mean±SD	87.18±7.72		87.39±7.39		0.651^b^
≤ 85	253	44.7	249	44.0	
85–92	109	19.3	101	17.8	
> 92	204	36.0	216	38.2	
High frequency hearing threshold shift (dB)					**<0.001**^a^
Mean±SD	35.77±9.86		14.02±4.17		**<0.001**^b^
≤ 26	59	10.4	566	100.0	
> 26	507	89.6	0	0.0	

^a^ Two-sided χ^2^ test

^b^ *Students’* t-test.

**Table 2:** General information of selected SNPs and Hardy-Weinberg test.

SNP	Alleles	Chromosome	Functional Consequence	MAF		*P* for HWE ^b^
				Control	Database ^a^	
rs2802292	G/T	6:108587315	5’ UTR variant	0.212	0.189	0.172
rs10457180	A/G	6:108643836	Intron variant	0.205	0.200	0.754
rs12206094	C/T	6:108584997	Intron variant (upstream variant 2KB)	0.143	0.146	0.858

^a^ Data from NCBI dbSNP

^b^ *P* value of Hardy-Weinberg test.

### Multivariate analyses of FOXO3 SNPs with the risk of NIHL

Three FOXO3 SNPs (rs2802292, rs10457180, and rs12206094) were selected to genotype in 1132 noise exposed workers (566 NIHL patients and 566 controls). **Table 3** presents the results of genotype and allele distributions of rs2802292, rs10457180, and rs12206094 in codominant, dominant, recessive and allelic model. Results are shown that the genotype frequencies of rs2802292, rs10457180, and rs12206094 in codominant model among cases and controls were statistically significantly different (*P* = 0.001, 0.001 and 0.046 respectively). In the dominant model, the rs2802292 GT+GG and rs10457180 AG+GG were significantly associated with NIHL risk (*P* = 0.008 and 0.005 respectively). Subsequent logistic regression analysis adjusting for age, sex, smoking and drinking showed that individuals with rs2802292 GT+GG and rs10457180 AG+GG had increased NIHL risk with OR of 1.40 (95%CI = 1.10–1.78) and 1.44 (95%CI = 1.13–1.82). In the recessive model, the rs2802292 GG (OR = 2.24, 95% CI = 1.38–3.36), rs10457180 GG (OR = 2.16, 95% CI = 1.29–3.61) and rs12206094 TT (OR = 2.10, 95% CI = 1.04–4.24) genotypes conferred a significantly increased risk for NIHL (*P* = 0.001, 0.004, and 0.043 respectively). In the allelic model, the rs2802292 G (OR = 1.43, 95% CI = 1.18–1.74), rs10457180 G (OR = 1.43, 95% CI = 1.18–1.75) and rs12206094 T (OR = 1.31, 95% CI = 1.04–1.64) alleles conferred a significantly increased risk for NIHL (*P* <0.001, *P* <0.001, and *P* = 0.026 respectively). Thus, our data revealed that FOXO3 SNP rs2802292, rs10457180, and rs12206094 may have a significant association with NIHL risk, and those individuals who carry rs2802292 G, rs10457180 G, and rs12206094 T allele may have significantly increased NIIHL susceptibility.

**Table 3:** Distribution of three polymorphisms and the association with NIHL.

Genetic models	Genotypes	Cases		Controls		*P*^a^	Adjusted OR
		n = 566	%	n = 566	%		(95% CI)^b^
rs2802292							
Codominant	TT	307	54.2	352	62.2	**0.001**	1.00 (Ref.)
	GT	204	36.0	188	33.2		1.26 (0.98–1.02)
	GG	55	9.7	26	4.6		**2.44 (1.49–4.00)**
Dominant	TT	307	54.2	352	62.2	**0.008**	1.00 (Ref.)
	GT/GG	259	45.8	214	37.8		**1.40 (1.10–1.78)**
Recessive	TT/GT	511	90.3	540	95.4	**0.001**	1.00 (Ref.)
	GG	55	9.7	26	4.6		**2.24 (1.38–3.63)**
Alleles	T	818	72.3	892	78.8	<**0.001**	1.00 (Ref.)
	G	314	27.7	240	21.2		**1.43 (1.18–1.74)**
rs10457180							
Codominant	AA	309	54.6	357	63.1	**0.001**	1.00 (Ref.)
	AG	210	37.1	186	32.9		**1.32 (1.03–1.69)**
	GG	47	8.3	23	4.1		**2.40 (1.42–4.05)**
Dominant	AA	309	54.6	357	63.1	**0.005**	1.00 (Ref.)
	AG/GG	257	45.4	209	36.9		**1.44 (1.13–1.82)**
Recessive	AA/AG	519	91.7	543	95.9	**0.004**	1.00 (Ref.)
	GG	47	8.3	23	4.1		**2.16 (1.29–3.61)**
Alleles	A	828	73.1	900	79.5	<**0.001**	1.00 (Ref.)
	G	304	26.9	232	20.5		**1.43 (1.18–1.75)**
rs12206094							
Codominant	CC	389	68.7	416	73.5	**0.046**	1.00 (Ref.)
	CT	152	26.9	138	24.4		1.19 (0.91–1.56)
	TT	25	4.4	12	2.1		**2.20 (1.09–4.45)**
Dominant	CC	389	68.7	416	73.5	0.088	1.00 (Ref.)
	CT/TT	177	31.3	150	26.5		1.28 (0.99–1.65)
Recessive	CC/CT	541	95.6	554	97.9	**0.043**	1.00 (Ref.)
	TT	25	4.4	12	2.1		**2.10 (1.04–4.24)**
Alleles	C	930	82.2	970	85.7	**0.026**	1.00 (Ref.)
	T	202	17.8	162	14.3		**1.31 (1.04–1.64)**

^a^ Two-sided χ^2^ test

^b^ Adjusted for age, sex, smoking, drinking in logistic regression model.

### Stratified analyses of rs2802292, rs10457180, and rs12206094 polymorphism and NIHL risk

The impacts of rs2802292, rs10457180, and rs12206094 genotypes in NIHL on a series of risk characteristics were analyzed in a dominant model. The results were shown in **Table 4**. Significant differences were found in the genotype distributions between cases and controls for the group in noise exposure time (>16 years) in rs2802292 and rs10457180 (*P* = 0.006 and 0.004). After adjusting for age, sex, smoking, and drinking in logistic regression model, an increased NIHL risk was found in the subjects who exposed to noise >16 years with rs2802292 GG/GT and rs10457180 AG/GG genotype with an OR of 1.62 (95% CI = 1.17–2.26) and 1.66 (95% CI = 1.19–2.31). For persons exposed to noise ≤85dB with rs10457180 AG/GG have an increased risk for NIHL (OR = 1.81, 95% CI = 1.25–2.61, *P* = 0.003).

**Table 4:** Stratified analysis of SNPs in a dominant model.

SNPs	Group	Genotype	Work time with noise (years)		Expose level with noise (dB)		
			≤16	> 16	≤85	85–92	> 92
rs2802292	case	GG/GT	113	146	112	52	95
		TT	135	172	141	57	109
	control	GG/GT	107	107	89	33	92
		TT	154	198	160	68	124
	*P*^a^		0.298	**0.006**	0.051	0.027	0.413
	Adjusted OR (95% CI)^b^		1.22 (0.85–1.74)	**1.62 (1.17–2.26)**	1.50 (1.04–2.16)	1.77 (1.00–3.13)	1.18 (0.80–1.75)
rs10457180	case	AG/GG	115	142	117	49	91
		AA	133	176	136	60	113
	control	AG/GG	107	102	83	32	94
		AA	154	203	166	69	122
	*P*^a^		0.222	**0.004**	**0.003**	0.048	0.822
	Adjusted OR (95% CI)^b^		1.27 (0.89–1.81)	**1.66 (1.19–2.31)**	**1.81 (1.25–2.61)**	1.73 (0.97–3.09)	1.06 (0.72–1.57)
rs12206094	case	CT/TT	80	97	77	33	67
		CC	168	221	176	76	137
	control	CT/TT	78	72	62	18	70
		CC	183	233	187	83	146
	*P*^a^		0.563	0.053	0.166	0.035	0.924
	Adjusted OR (95% CI)^b^		1.14 (0.78–1.66)	1.47 (1.02–2.10)	1.39 (0.93–2.07)	1.87 (0.96–3.65)	1.02 (0.67–1.54)

^a^ Two-sided χ^2^ test.

^b^ Adjusted for age, sex, smoking, drinking in logistic regression model.

### Association between the haplotypes of FOXO3 SNPs with NIHL risk

Furthermore, analysis of the haplotype frequencies of the three SNPs was performed between NIHL cases and controls (**Table 5**). Four common haplotypes (frequency > 5%) derived from the three SNPs accounting for 90% of the haplotype variations were selected and the rest of haplotypes were pooled into the mixed group. Ultimately, the haplotype GAC and others (TGT/GGT/GGC/GAT) (rs2802292-rs10457180-rs12206094) in FOXO3 gene were found to be associated with an increased risk for NIHL (OR = 1.49 and 2.09) compared to TAC haplotype.

**Table 5:** Frequencies of inferred haplotypes among the cases and controls and their association with risk NIHL.

Haplotypes^a^	Case (n = 566)		Control (n = 566)		*P*^b^	Adjusted OR	Global *P*^*d*^
	n	%	n	%		(95% CI)^c^	
TAC	610	53.9	697	61.6		1.00 (Ref.)	**<0.001**
GAC	209	18.5	190	16.8	**0.039**	**1.27 (1.01–1.59)**	
TGT	130	11.5	118	10.4	0.080	1.28 (0.97–1.68)	
TGC	72	6.4	66	5.8	0.209	1.25 (0.88–1.78)	
Others^e^	111	9.8	61	5.4	**<0.001**	**2.09 (1.50–2.91)**	

^a^ The alleles of haplotypes were arrayed as rs2802292-rs10457180-rs12206094.

^b^ Two-sided χ^2^ test.

^c^ Adjusted for age, sex, smoking, drinking in logistic regression model.

^d^ Generated by permutation test with 1000 times of simulation.

^e^ Haplotypes (TGT/GGT/GGC/GAT) were pooled into the mixed group.

### Comparison of high-frequency hearing threshold shift of three SNPs genotypes

**Fig 1:**
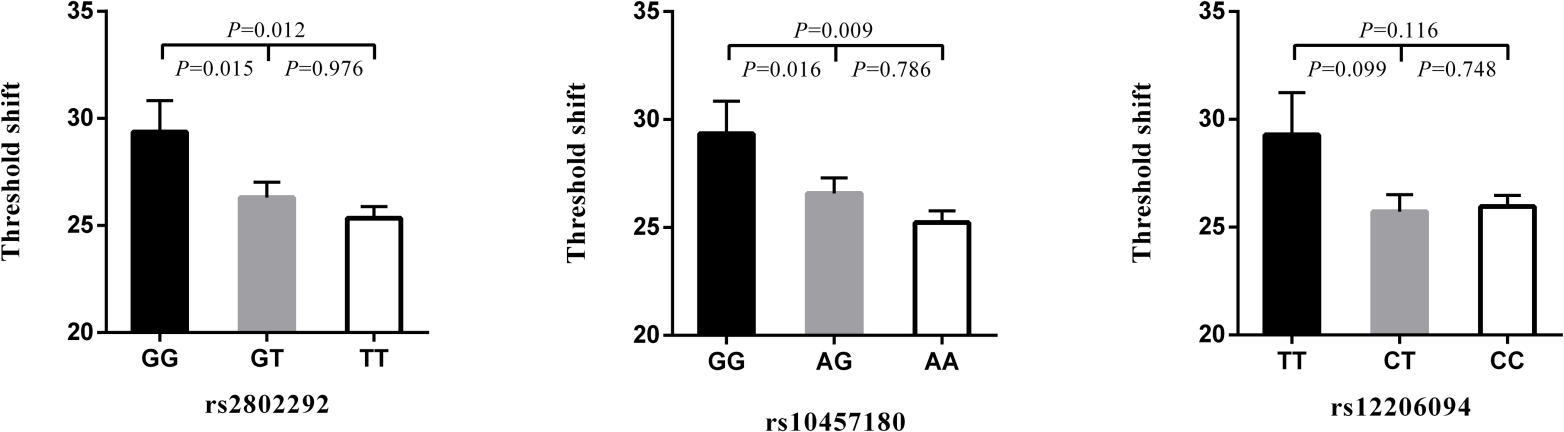
Comparison of high-frequency hearing threshold shift of three SNPs. Comparison of high-frequency hearing threshold shift of rs2802292, rs10457180, and rs12206094 genotypes in all subjects. Data are presented as mean ± SE and analyzed by ANOV.

**Fig 1** shows the results of the comparison of high-frequency hearing threshold shift of rs2802292, rs10457180, and rs12206094 genotypes in 1132 noise exposed workers. Subjects with GG genotype of rs2802292 have a significantly higher high-frequency hearing threshold shift than people with GT and TT genotype (*P* = 0.015 and 0.012). Persons with GG genotype of rs10457180 were also found to have higher high-frequency hearing threshold shift than people with AG and AA genotype (*P* = 0.016 and 0.009).

### Multifactor dimensionality reduction analyses of the interaction between the three SNPs

MDR analysis results of the interaction between the three SNPs were presented in **Table 6**. The interaction results suggested that the rs10457180-rs2802292-rs12206094 model got the highest cross-validation consistency and testing balanced accuracy and was related to increased NIHL risk (OR = 1.53, 95%CI = (1.20–1.94), *P* = 0.0005).

**Table 6:** MDR analysis results of the interaction between the three SNPs.

Model	Training balanced accuracy	Testing balanced accuracy	Cross-validation consistency	*P*	OR(95%CI)
rs10457180	0.5426	0.538	9/10	0.0037	1.42(1.12–1.80)
rs10457181-rs2802292	0.5463	0.5274	7/10	0.0016	1.47(1.16–1.87)
rs10457181-rs2802292-rs12206094	0.5507	0.538	10/10	0.0005	1.53(1.20–1.94)

## Discussion

Single nucleotide polymorphism (SNP) is the one of the most common types of genetic variant in the human genome and there are about fifteen million SNPs in all human group[21]. Haplotype, defined as a specific set of alleles observed on a single chromosome, or a part of a chromosome, has been an integral part of human genetics for decades[22]. However, the genomic distribution of SNPs is not homogenous; most of the SNPs occur in noncoding regions of gene more frequently than in coding regions. At present, detection methods of SNP include denaturing gradient gel electrophoresis (DDGE), single-strand conformational polymorphism (SSCP), cleaved amplified polymorphic sequence (CAPS), denaturing gradient gel electrophoresis (DDGE), and allele-specific PCR (such as TaqMan SNP genotype-PCR).

In the current study, a genetic association analysis was performed on three selected FOXO3 SNPs (rs2802292, rs10457180, and rs12206094) in 566 NIHL patients and 566 controls by TaqMan SNP genotyping assay. Results reveal that rs2802292 G allele, rs10457180 G allele, and rs12206094 T allele in FOXO3 are associated with a significantly higher risk of NIHL. Subsequent haplotype analysis shows that the haplotype GAC and others (TGT/GGT/GGC/GAT) (rs2802292-rs10457180-rs12206094) confers increased risks of NIHL. These findings support our hypothesis that the FOXO3 polymorphism may contribute to susceptibility to NIHL. As far as we know, this may be the first association study showing that FOXO3 gene is correlated with an increased risk of NIHL in a Chinese population.

Recent research has shown the essential role of transcription factor FOXO3 in the cochlea in hearing maintenance[17]. Foxo3 protein is detected in the nuclei of spiral ganglion neurons, hair cells, and pillar cells of young adult mice suggesting that it acts in sensory cells. Notably, Foxo3 protein becomes localized to the cytoplasm of spiral ganglion neurons as mice age. Exposure to nondamaging noise levels drives Foxo3 nuclear localization indicating that higher cochlear activity levels promote Foxo3-dependent transcription by increasing Foxo3 concentration near DNA[17]. Moreover, Foxo3 can drive transcription of the master mitochondrial regulator Pgc-1α. Pgc-1α coordinates new production of mitochondrial proteins from both the nucleus and mitochondrial DNA (mtDNA)[23]. In conjunction with Pgc-1α, Foxo3 directly interacts with the promoters of Sod2, catalase, and peroxiredoxin to drive their expression[23]. Sod2, catalase, and peroxiredoxin which contains free radicals have been known to have an essential role in the cochlea in noise-induced hearing loss (NIHL)[5]. Foxo3 is required for auditory function after noise exposure in a mouse model system and absent Foxo3, outer hair cells are lost throughout the middle and higher frequencies[18]. Above evidence support an influential role of transcription factor Foxo3 in contributing to the maintenance and protection of hearing.

Despite all the three SNPs selected locate in noncoding region of FOXO3 gene, numerous studies have shown that functional consequences of noncoding SNPs involve in the regulation of protein-coding genes and lncRNA regulation[24, 25]. Genetic variation at FOXO3 rs12212067 has been proved to drive allele-specific expression of FOXO3 and modulate inflammatory cytokine production in monocytes through a paracrine TGFβ1-dependent mechanism[26]. Also, Foxo3 intron variations may alter the dysregulated biogenesis of circ-Foxo3. Ectopic expression of circ-Foxo3 could bind to the cell cycle proteins cyclin-dependent kinase 2 and cyclin-dependent kinase inhibitor 1, resulting in the formation of a ternary complex which arrests the function of CDK2 and blocked cell cycle progression[27].

Our current study has several potential limitations. (i) The sample size of our study was relatively larger compared to previous research. However, the power of statistical tests may not be fully enough due to the lower biological effects of an individual SNP. Therefore, large sample size and cohort studies are needed in future to confirm the effects of the FOXO3 polymorphism on NIHL; (ii) Study subjects of this case-control study are limited by the Chinese population. Thus, our results may likely be better generalized to Chinese Han population and limit external generalizability; (iii) The selected SNPs are noncoding, thus we assume these variants to be linked with one or more functional variants within the FOXO3 gene or its regulatory regions. Future fine mapping of the FOXO3 gene may detect such functional variants; (iv) The loss of connections between inner hair cells and auditory nerve has been proved to be associated with loss of FOXO3 by animal studies and could change suprathresholds audition. This "hidden hearing loss" is not always detected with the routine tests of changes in hearing thresholds by pure tone audiometry. As this is a cohort study, suprathreshold audiometry is necessary in the follow-up examination of these noise exposed workers to detect "hidden hearing loss" and verify the role of FOXO3 gene variation in noise induced hearing loss.

In conclusion, rs2802292 G allele, rs10457180 G allele, and rs12206094 T allele in FOXO3 are associated with a significantly higher risk of NIHL. These three noncoding SNPs may involve in the regulation of protein-coding genes, lncRNA, and circRNA mechanism of FOXO3. Thus, these findings show that FOXO3 SNPs (rs2802292, rs10457180, and rs12206094) may have important roles in noise induced hearing loss and have potential to be biomarkers for noise exposed workers.

## Supporting information

S1 File Information of each subject. Demographic characteristics and genotype result of each subject.
(XLS)
